# Supplementary material for: Promoting leisure functions through setting creative linguistic landscapes in recreational zones
Source: PLoS One. 2024 Mar 22;19(3):e0299775. doi: 10.1371/journal.pone.0299775 (PMC10959382; doi:10.1371/journal.pone.0299775)
Supplement: S1 Questionnaire — (DOCX) [file pone.0299775.s002.docx]

***Please confirm**: this questionnaire is voluntary. After filling out, it is deemed that the information in it can be used for research on *Promoting leisure functions through setting creative linguistic landscapes in recreational zones*.

**A Functional Investigation of the Creative Linguistic Landscapes**

There are about 9 types of Creative Linguistic Landscapes(CLL) on the market, namely: ①Emotion exchanging, ②Creating atmosphere, ③Mood regulation, ④Mentality guidance, ⑤Catering to wishes, ⑥Inducing behaviors, ⑦Labeling site, ⑧Building cognition, ⑨Resonating with movie lines. For each type of CLL, there are selected three representative images and presented them on the 30×10 cm color photograph; participants are asked to rank the corresponding functional impact of each type of CLL for every function indicator; researchers work one-on-one with each participant to complete the ranking.

**There are 9 groups of pictures on the 30×10 cm color photograph (each group has 3 representative pictures) showing these 9 types of Creative Linguistic Landscapes. Please assist us in completing the following research questions based on your multiple tastings of the color photograph:**

1. Among the above 9 groups of Creative Linguistic Landscapes, the degree of ***Producing innovative content*** presented by each type of linguistic landscapes, in descending order, are as follows: (please use 1, 2, 3, 4, 5, 6, 7, 8 and 9 to sort)

、 、 、 、 、 、 、 、

2. Among the above 9 groups of Creative Linguistic Landscapes, the degree of ***Manifesting a unique image*** presented by each type of linguistic landscapes, in descending order, are as follows: (please use 1, 2, 3, 4, 5, 6, 7, 8 and 9 to sort)

、 、 、 、 、 、 、 、

3. Among the above 9 groups of Creative Linguistic Landscapes, the degree of ***The limit of suitable locations*** presented by each type of linguistic landscapes, in descending order, are as follows: (please use 1, 2, 3, 4, 5, 6, 7, 8 and 9 to sort)

、 、 、 、 、 、 、 、

4. Among the above 9 groups of Creative Linguistic Landscapes, the degree of ***Not easy to update*** presented by each type of linguistic landscapes, in descending order, are as follows: (please use 1, 2, 3, 4, 5, 6, 7, 8 and 9 to sort)

、 、 、 、 、 、 、 、

5. Among the above 9 groups of Creative Linguistic Landscapes, the degree of ***Easy to become outdated*** presented by each type of linguistic landscapes, in descending order, are as follows: (please use 1, 2, 3, 4, 5, 6, 7, 8 and 9 to sort)

、 、 、 、 、 、 、 、

6. Among the above 9 groups of Creative Linguistic Landscapes, the degree of ***Enhancing visitors’ experiences*** presented by each type of linguistic landscapes, in descending order, are as follows: (please use 1, 2, 3, 4, 5, 6, 7, 8 and 9 to sort)

、 、 、 、 、 、 、 、

7. Among the above 9 groups of Creative Linguistic Landscapes, the degree of ***Adjusting visitors’ mood*** presented by each type of linguistic landscapes, in descending order, are as follows: (please use 1, 2, 3, 4, 5, 6, 7, 8 and 9 to sort)

、 、 、 、 、 、 、 、

8. Among the above 9 groups of Creative Linguistic Landscapes, the degree of ***Promoting emotional expression*** presented by each type of linguistic landscapes, in descending order, are as follows: (please use 1, 2, 3, 4, 5, 6, 7, 8 and 9 to sort)

、 、 、 、 、 、 、 、

9. Among the above 9 groups of Creative Linguistic Landscapes, the degree of ***Promoting negative experiences*** presented by each type of linguistic landscapes, in descending order, are as follows: (please use 1, 2, 3, 4, 5, 6, 7, 8 and 9 to sort)

、 、 、 、 、 、 、 、

10. Among the above 9 groups of Creative Linguistic Landscapes, the degree of ***Obstructing recreational activities*** presented by each type of linguistic landscapes, in descending order, are as follows: (please use 1, 2, 3, 4, 5, 6, 7, 8 and 9 to sort)

、 、 、 、 、 、 、 、

11. Among the above 9 groups of Creative Linguistic Landscapes, the degree of ***Promoting online marketing*** presented by each type of linguistic landscapes, in descending order, are as follows: (please use 1, 2, 3, 4, 5, 6, 7, 8 and 9 to sort)

、 、 、 、 、 、 、 、

12. Among the above 9 groups of Creative Linguistic Landscapes, the degree of ***Attracting attention on site*** presented by each type of linguistic landscapes, in descending order, are as follows: (please use 1, 2, 3, 4, 5, 6, 7, 8 and 9 to sort)

、 、 、 、 、 、 、 、
